# Evaluation of the Admission Neutrophil Percentage-to-Albumin Ratio for Predicting the Severity of Acute Pancreatitis

**DOI:** 10.3390/biomedicines14071543

**Published:** 2026-07-09

**Authors:** Ahmet Yavuz, Berat Ebik, Ümit Karabulut, Mustafa Zanyar Akkuzu, Çiğdem Budak Ece, Ferhat Bacaksız, Muharrem Keskin, Murat Bıyık, Mehmet Asıl

**Affiliations:** 1 Department of Gastroenterology, Diyarbakır Gazi Yasargil Education and Research Hospital, University of Health Sciences, Diyarbakır 21070, Turkey; beratebik@gmail.com (B.E.); drumitkarabulut@gmail.com (Ü.K.); zanyarakkuzu@gmail.com (M.Z.A.); cgdmbdk@hotmail.com (Ç.B.E.); feratson85@hotmail.com (F.B.); 2 Department of Gastroenterology, Faculty of Medicine, Necmettin Erbakan University, Konya 42090, Turkey; muharremkeskin@gmail.com (M.K.); drmuratbiyik@gmail.com (M.B.); drmehmetasil@yahoo.com.tr (M.A.)

**Keywords:** Acute Pancreatitis (AP), neutrophil percentage-to-albumin ratio (NPAR), inflammatory index, disease severity

## Abstract

**Background:** Early identification of disease severity in acute pancreatitis (AP) remains a major clinical challenge. The neutrophil percentage-to-albumin ratio (NPAR) is a novel inflammatory index that reflects systemic inflammation, but evidence regarding its role in predicting AP severity remains limited. This study aimed to evaluate the association between admission NPAR and AP severity and to compare its predictive performance with other inflammatory indices. **Methods:** This retrospective study included 261 patients with AP. NPAR, neutrophil-to-lymphocyte ratio (NLR), platelet-to-lymphocyte ratio (PLR), and the systemic immune inflammation index (SII) were calculated using laboratory parameters obtained at hospital admission. Disease severity was assessed according to the revised Atlanta classification and the Bedside Index for Severity in Acute Pancreatitis (BISAP) score. Receiver operating characteristic (ROC) curve analysis was performed to compare predictive performance, and logistic regression analyses were used to identify independent predictors of disease severity. **Results:** Admission NPAR values were significantly higher in patients with moderately severe/severe AP according to the revised Atlanta classification and in patients with BISAP scores ≥ 3 (both *p* < 0.001). Among the evaluated inflammatory indices, NPAR demonstrated the highest predictive performance for disease severity according to both the revised Atlanta classification (AUC: 0.808) and BISAP score (AUC: 0.841). In multivariate logistic regression analysis, admission NPAR remained independently associated with AP severity (OR: 1.279, 95% CI: 1.174–1.393, *p* < 0.001). Admission NPAR levels were also significantly higher in non-survivors than in survivors (*p* = 0.017). **Conclusions:** Admission NPAR appears to be a simple, inexpensive, and readily available inflammatory index associated with disease severity in patients with AP. These findings suggest that NPAR may serve as a useful adjunctive tool for early risk stratification. However, larger prospective multicenter studies are required to validate these findings and confirm the proposed admission cut-off value before routine clinical implementation.

## 1. Introduction

Acute pancreatitis (AP) is an inflammatory disease of the pancreas that develops following injury to pancreatic acinar cells and may lead to both local pancreatic damage and systemic inflammatory responses. The reported annual incidence of AP ranges between 5 and 100 cases per 100,000 individuals worldwide [1]. Based on the revised Atlanta Classification, AP is categorized into three severity groups: mild, moderately severe, and severe. Mild AP is characterized by the absence of organ failure and complications, whereas moderately severe AP includes transient organ failure lasting less than 48 h and/or local or systemic complications. Severe AP is defined by persistent organ failure affecting one or more organs [2].

Most patients experience a mild clinical course, and the disease usually resolves within one week with appropriate supportive treatment. Nevertheless, approximately 20% of patients develop moderately severe or severe AP, which may be associated with local complications, systemic inflammatory responses, and significantly increased mortality. In patients with severe AP, mortality rates exceeding 20% have been reported [3].

Because severe AP is associated with substantial morbidity and mortality, early identification of high-risk patients is of considerable clinical importance. For this purpose, several scoring systems have been developed to estimate disease severity. Among the most commonly used are the Ranson criteria, the Harmless Acute Pancreatitis Score (HAPS), the Acute Physiology and Chronic Health Evaluation II (APACHE II), the Systemic Inflammatory Response Syndrome (SIRS) criteria, the Bedside Index of Severity in Acute Pancreatitis (BISAP), and Imrie’s score (modified Glasgow score). However, none of these scoring systems has proven to be ideal for routine clinical use. For example, the Ranson criteria require a 48 h observation period before all variables can be evaluated, which limits their usefulness for early decision-making [4]. Similarly, although APACHE II is widely applied, it requires numerous clinical and laboratory variables and may be difficult to calculate in daily clinical practice [5].

Due to these limitations, recent research has focused on identifying simple and easily obtainable biomarkers that could help predict disease severity in patients with AP. Several studies have shown that indices derived from routine hematological parameters, such as the neutrophil-to-lymphocyte ratio (NLR), platelet-to-lymphocyte ratio (PLR), and the Systemic Immune Inflammation Index (SII), may provide useful information regarding the severity and prognosis of AP [6,7].

Neutrophils are known to play a key role in the inflammatory cascade associated with AP. In the early stages of the disease, neutrophils migrate into pancreatic tissue and release proteolytic enzymes, reactive oxygen species, and pro-inflammatory mediators. These processes contribute to tissue injury and may promote systemic inflammatory responses. Consequently, increased neutrophil counts and percentages have been associated with more severe disease in patients with AP [8].

Albumin is an important plasma protein that contributes to the maintenance of oncotic pressure and also exerts antioxidant and anti-inflammatory effects. In inflammatory conditions and states of malnutrition, serum albumin levels tend to decrease. Previous studies have indicated that hypoalbuminemia may serve as an independent predictor of disease severity and mortality in patients with AP [9].

The neutrophil percentage-to-albumin ratio (NPAR), which combines neutrophil percentage and serum albumin into a single index, has recently emerged as a promising inflammatory biomarker reflecting both systemic inflammation and nutritional status. Previous studies have demonstrated the prognostic value of NPAR in a variety of inflammatory and critical illnesses, including sepsis, spontaneous bacterial peritonitis, cardiogenic shock, chronic kidney disease, metabolic dysfunction-associated steatotic liver disease (MASLD), and several malignancies [10,11,12,13,14,15]. More recently, NPAR has also been investigated as a diagnostic and prognostic biomarker across a broad spectrum of inflammatory and neoplastic disorders, further supporting its potential clinical applicability [16].

Evidence regarding the clinical utility of NPAR in AP remains limited. A recent cohort study conducted in critically ill patients with AP admitted to the intensive care unit demonstrated that elevated admission NPAR was associated with increased mortality [17]. However, whether admission NPAR can predict disease severity in an unselected population of patients presenting with AP has not been adequately investigated. Moreover, comparisons between NPAR and other readily available inflammatory indices, such as NLR, PLR, SII, remain scarce.

Therefore, the present study aimed to evaluate the association between admission NPAR and AP severity, compare its predictive performance with other readily available inflammatory indices, and determine whether NPAR is an independent predictor of disease severity.

## 2. Materials and Methods

### 2.1. Study Design and Population

In this study, data from patients who were followed and treated for AP in the gastroenterology clinic of our hospital between January 2019 and October 2023 were retrospectively reviewed. Ethical approval for the study was obtained from the local ethics committee of Health Sciences University Gazi Yaşargil Training and Research Hospital on 19 April 2024 (approval number: 10).

Patients aged 18 years and older who were followed in our clinic with a diagnosis of AP were included in the study. Patients with diseases causing chronic inflammation, chronic pancreatitis, pregnancy, those receiving immunosuppressive therapy, patients with liver failure, and those who had received albumin replacement therapy were excluded from the study. Patients with concomitant acute cholecystitis, cholangitis, or other acute infections were excluded because these conditions may independently alter inflammatory indices, particularly neutrophil-related parameters, thereby potentially confounding the assessment of inflammation associated with AP.

**Figure 1:**
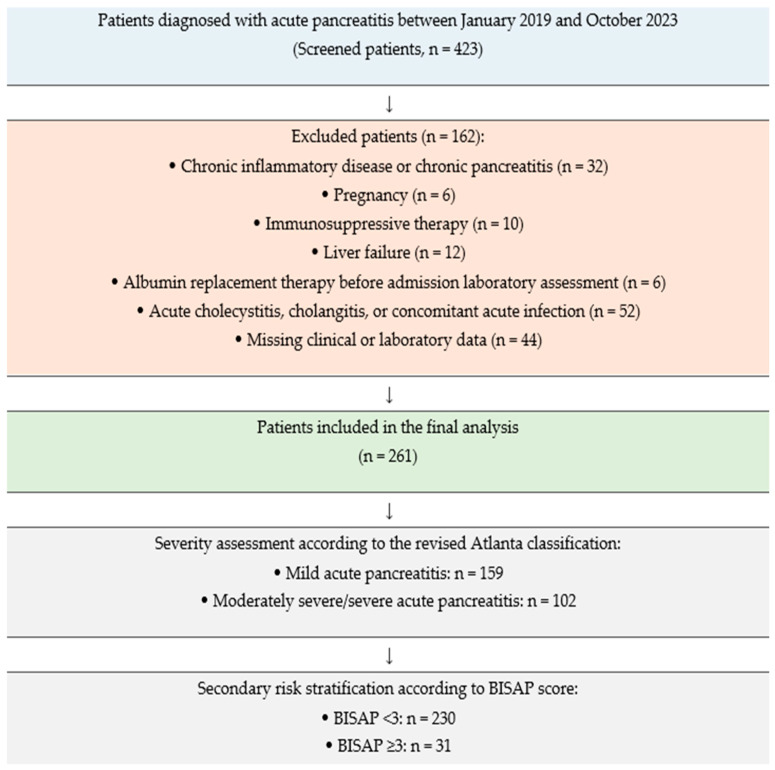
Patient selection flow diagram.

A total of 261 patients were included in the study after application of the predefined inclusion and exclusion criteria (Figure 1). Data on age, sex, etiology of AP, accompanying comorbidities, length of hospital stay, mortality, complete blood count, C-reactive protein (CRP), urea, creatinine, albumin, calcium, aspartate aminotransferase (AST), alanine aminotransferase (ALT), lactate dehydrogenase (LDH), amylase, and lipase levels were collected from the electronic medical records. All laboratory parameters used in the analyses were obtained from blood samples collected at hospital admission before initiation of treatment. Imaging findings, including ultrasonography (US), computed tomography (CT), and magnetic resonance imaging (MRI), were also evaluated.

### 2.2. Diagnosis and Clinical Management

The diagnosis of AP was established when at least two of the following three criteria were present: (1) typical abdominal pain characterized by sudden-onset epigastric pain radiating to the back in a belt-like pattern; (2) an elevation of serum amylase or lipase levels to at least three times the upper limit of normal; and (3) imaging findings consistent with AP on US, CT, or MRI [2].

In our clinic, the treatment of AP is performed in accordance with the recommendations of international guidelines. After a definitive diagnosis is established, appropriate intravenous hydration and analgesic therapy are administered for pain control. Oral intake is discontinued if the patient has pain; however, as pain decreases and if tolerated by the patient, oral feeding is resumed as early as possible, generally within the first 24 h. Routine antibiotic therapy is not administered in AP unless there is evidence of a documented infection.

### 2.3. Severity Classification

Patients were evaluated according to the revised Atlanta Classification as having mild (no organ failure and no local or systemic complications), moderately severe (transient organ failure resolving within 48 h and/or local or systemic complications without persistent organ failure), or severe AP (persistent organ failure involving one or more organs). Organ failure was defined according to the Modified Marshall scoring system and included cardiovascular failure (systolic blood pressure < 90 mmHg despite adequate fluid resuscitation), respiratory failure (PaO_2_ ≤ 60 mmHg), and renal failure (serum creatinine > 2 mg/dL). The presence of any of the following findings on imaging—acute fluid collection, pancreatic necrosis, or pancreatic abscess—was considered a local complication [2]. For statistical analysis, patients were grouped as mild AP and moderately severe/severe AP according to the revised Atlanta classification.

BISAP scores were calculated for all patients within the first 24 h based on the following criteria: blood urea nitrogen > 25 mg/dL, impaired mental status, SIRS, age > 60 years, and pleural effusion [18]. As a secondary analysis, patients were additionally stratified according to the BISAP score (<3 vs. ≥3), as this threshold has been widely used to identify patients at increased risk for severe AP and adverse clinical outcomes [1,18].

### 2.4. Calculation of Inflammatory Indices

NLR was calculated as the absolute neutrophil count divided by the absolute lymphocyte count. PLR was calculated as the platelet count divided by the absolute lymphocyte count. SII was calculated as the platelet count × neutrophil count divided by the absolute lymphocyte count. NPAR was calculated as the neutrophil percentage (%) divided by the serum albumin concentration (g/dL).

### 2.5. Statistical Analysis

Statistical analyses were performed using IBM SPSS Statistics for Windows, version 24.0 (IBM Corp., Armonk, NY, USA). The normality of the data was assessed using the Kolmogorov–Smirnov and Shapiro–Wilk tests, as well as skewness and kurtosis values. Continuous variables were presented as mean ± standard deviation or median (minimum–maximum), whereas categorical variables were expressed as numbers and percentages. Categorical variables were compared using the chi-square test (χ^2^) or Fisher’s exact test, as appropriate. Comparisons between independent patient groups were performed using the Independent Samples t-test for normally distributed variables and the Mann–Whitney U test for non-normally distributed variables. The Mann–Whitney U test was also used to compare NPAR values between survivors and non-survivors.

Receiver operating characteristic (ROC) curve analysis was performed to evaluate the predictive performance of NPAR, NLR, PLR, and SII for moderately severe/severe AP according to the revised Atlanta classification and for patients with BISAP scores ≥ 3. Areas under the curve (AUCs) were calculated for all inflammatory indices. In addition, the optimal cut-off value, sensitivity, specificity, positive predictive value (PPV), and negative predictive value (NPV) were determined for NPAR.

Univariate logistic regression analysis was initially performed to identify variables associated with moderately severe/severe AP according to the revised Atlanta classification. Variables with a *p* value < 0.10 in the univariate analysis were considered candidates for multivariable logistic regression. To reduce the risk of overfitting, the final multivariable model included a limited number of variables selected based on statistical significance and established clinical relevance. Odds ratios (ORs) with 95% confidence intervals (CIs) were calculated. Model calibration was assessed using the Hosmer–Lemeshow goodness-of-fit test, and the explanatory power of the model was evaluated using Nagelkerke’s R^2^.

Subgroup analyses were additionally performed to evaluate the association between admission NPAR and AP severity according to age (<60 vs. ≥60 years), sex, and the presence or absence of comorbidities (defined as the presence of at least one documented chronic comorbid condition). All statistical tests were two-sided, and a *p* value < 0.05 was considered statistically significant.

## 3. Results

A total of 261 patients met the eligibility criteria and were included in the final analysis. The mean age was 58.0 ± 18.6 years, and 55.2% of the patients were female. Biliary disease was the most common etiology of AP (75.5%). According to the revised Atlanta classification, 159 patients (60.9%) had mild AP and 102 (39.1%) had moderately severe/severe AP. In the secondary analysis based on the BISAP score, 230 patients (88.1%) had BISAP scores < 3 and 31 (11.9%) had BISAP scores ≥ 3. The median length of hospital stay was 6 days (range, 1–69), 33 patients (12.6%) required intensive care unit admission, and the overall mortality rate was 3.1% (Table 1).

According to the revised Atlanta classification, glucose, LDH, urea, WBC, and neutrophil levels were significantly higher in patients with moderately severe/severe AP compared with those with mild AP (*p* < 0.001). In contrast, albumin, lymphocyte, and calcium levels were significantly lower in patients with moderately severe/severe AP compared with those with mild disease (*p* < 0.01, *p* < 0.001, and *p* = 0.003, respectively). All inflammatory indices, including NLR, NPAR, PLR, and SII, were significantly higher in patients with moderately severe/severe AP than in those with mild AP (all *p* < 0.001). No statistically significant difference was observed in C-reactive protein (CRP) levels between the mild and moderately severe/severe groups (*p* = 0.322). ICU admission, length of hospital stay, and mortality were significantly higher in the moderately severe/severe AP group than in the mild AP group (*p* < 0.001) (Table 1 and Table 2).

**Figure 2:**
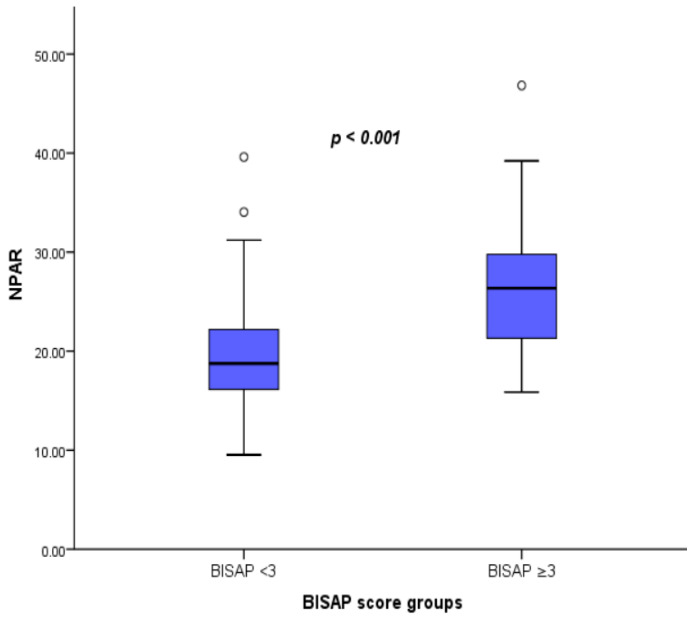
Admission NPAR levels according to BISAP score groups. Open circles indicate outliers.

Admission NPAR levels were significantly higher in patients with BISAP scores ≥ 3 than in those with BISAP scores < 3 (*p* < 0.001) (Figure 2).

**Figure 3:**
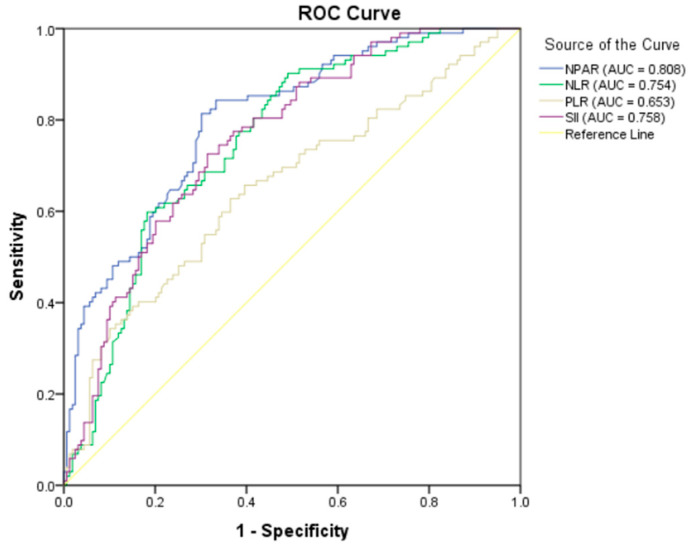
ROC curves comparing the performance of inflammatory indices for predicting moderately severe/severe AP according to the revised Atlanta classification.

ROC analysis showed that all inflammatory indices significantly predicted AP severity according to both the revised Atlanta classification and the BISAP score. According to the revised Atlanta classification, NPAR, NLR, SII, and PLR had AUC values of 0.808 (95% CI: 0.747–0.854, *p* < 0.001), 0.754 (95% CI: 0.696–0.813, *p* < 0.001), 0.758 (95% CI: 0.700–0.816, *p* < 0.001), and 0.653 (95% CI: 0.583–0.722, *p* < 0.001), respectively (Figure 3, Table 3).

**Figure 4:**
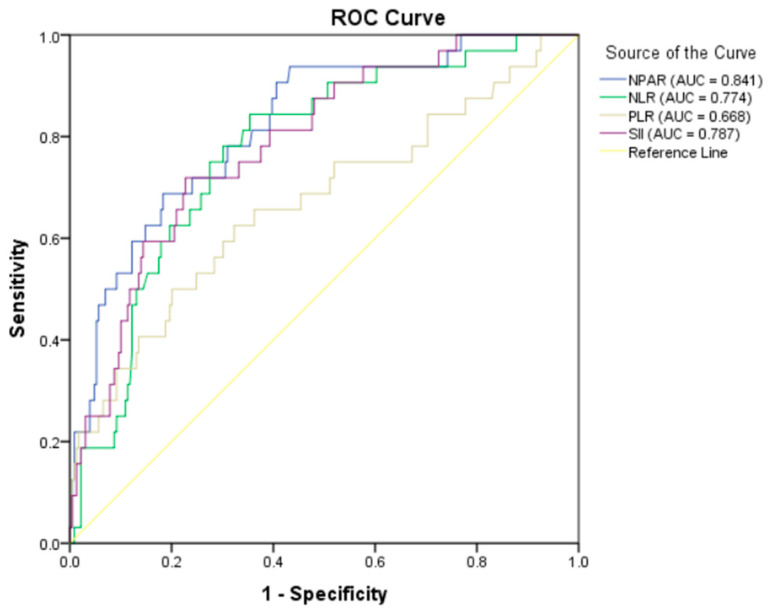
ROC curves comparing the performance of inflammatory indices for predicting BISAP ≥ 3.

In the BISAP-based analysis, the AUC values for NPAR, NLR, SII, and PLR were 0.841 (95% CI: 0.746–0.897, *p* < 0.001), 0.774 (95% CI: 0.693–0.855, *p* < 0.001), 0.787 (95% CI: 0.707–0.867, *p* < 0.001), and 0.668 (95% CI: 0.557–0.779, *p* = 0.002), respectively (Figure 4, Table 3).

Based on ROC analysis according to the revised Atlanta classification, the optimal admission NPAR cut-off value for predicting moderately severe/severe AP was 19.7, yielding a sensitivity of 81.6%, a specificity of 71.3%, a PPV of 63.1%, and an NPV of 85.1% (Table 4).

Univariate logistic regression analysis identified WBC, urea, and NPAR as significant predictors of moderately severe/severe AP according to the revised Atlanta classification (all *p* < 0.001), whereas age showed a borderline association (*p* = 0.095). In the multivariate logistic regression analysis, age (OR 0.968, 95% CI 0.949–0.988, *p* = 0.002), WBC (OR 1.133, 95% CI 1.041–1.233, *p* = 0.004), and NPAR (OR 1.279, 95% CI 1.174–1.393, *p* < 0.001) remained independently associated with disease severity, whereas urea was not (OR 1.017, 95% CI 0.998–1.037, *p* = 0.076). The multivariable model demonstrated good calibration according to the Hosmer–Lemeshow goodness-of-fit test (χ^2^ = 8.481, *p* = 0.388) and showed moderate explanatory power (Nagelkerke’s R^2^ = 0.417) (Table 5).

Subgroup analyses demonstrated that admission NPAR remained significantly higher in patients with moderately severe/severe AP than in those with mild disease across all evaluated subgroups, including age (<60 vs. ≥60 years), sex, and the presence or absence of comorbidities (all *p* < 0.001) (Supplementary Table S1).

**Figure 5:**
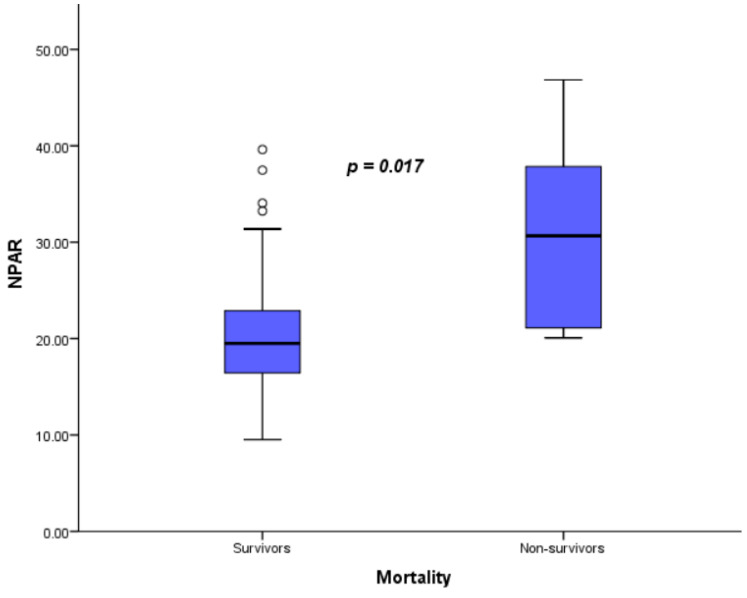
Comparison of admission NPAR values between survivors and non-survivors. Open circles indicate outliers.

Median NPAR levels were significantly higher in non-survivors compared with survivors (30.6 vs. 19.5, *p* = 0.017) (Figure 5).

## 4. Discussion

In the present study, we demonstrated that admission NPAR is a simple, inexpensive, and readily available inflammatory index associated with AP severity. Admission NPAR levels were significantly higher in patients with moderately severe/severe AP according to the revised Atlanta classification and in patients with BISAP scores ≥ 3. In ROC analysis, NPAR showed the highest predictive performance among the evaluated inflammatory indices (NLR, PLR, and SII), and it remained an independent predictor of disease severity in the multivariate logistic regression model. In addition, the association between elevated admission NPAR and disease severity remained consistent across predefined subgroups based on age, sex, and comorbidity status, and admission NPAR levels were also significantly higher in non-survivors. Collectively, these findings suggest that admission NPAR may serve as a useful adjunctive tool for early risk stratification in patients with AP.

In addition to NPAR, WBC was identified as an independent predictor of AP severity in the multivariate logistic regression analysis. In AP, activation of the inflammatory cascade leads to increased production of pro-inflammatory cytokines such as interleukin-6 (IL-6), tumor necrosis factor alpha (TNF-α), interleukin-8 (IL-8), interleukin-4 (IL-4), and transforming growth factor beta (TGF-β). These cytokines promote leukocyte activation, aggregation, and migration, resulting in an enhanced white blood cell response and contributing to both local pancreatic inflammation and systemic inflammatory effects [19,20,21,22]. Leukocytosis reflects the magnitude of the systemic inflammatory response and has long been recognized as an important component of initial severity assessment in AP. Elevated WBC levels are included in widely used prognostic scoring systems such as the Ranson criteria, APACHE II, and BISAP and have been associated with pancreatic necrosis, persistent organ failure, and increased mortality in previous studies [23,24,25].

Several inflammatory indices derived from routine hematological parameters, including NLR, PLR, SII, have been reported to predict disease severity, adverse clinical outcomes, and mortality in patients with AP [7,26,27]. In the present study, all three indices demonstrated significant predictive performance for AP severity in ROC analysis. However, NPAR exhibited the highest discriminative ability among the evaluated inflammatory indices and remained an independent predictor of disease severity in the multivariate logistic regression analysis. These findings suggest that integrating neutrophil percentage, which reflects the intensity of the inflammatory response, with serum albumin, an indicator of both systemic inflammation and nutritional status, provides a more comprehensive assessment of disease severity than hematological indices alone.

Serum albumin levels are frequently reduced in patients with AP. The mechanisms underlying hypoalbuminemia are multifactorial and include reduced hepatic albumin synthesis, increased vascular permeability with translocation of albumin into the interstitial space, enhanced protein catabolism, and the effects of pro-inflammatory cytokines such as IL-6 and TNF-α [28]. Furthermore, serum albumin concentrations may also be influenced by hydration status, nutritional status, and underlying chronic conditions, which should be taken into consideration when interpreting serum albumin levels in patients with AP. Previous studies have identified hypoalbuminemia as an independent predictor of AP severity, persistent organ failure, and mortality [9,29]. In the present study, serum albumin levels were significantly lower in patients with moderately severe/severe AP, consistent with previous reports. Neutrophils play a central role in the inflammatory cascade of AP by releasing proteolytic enzymes, reactive oxygen species, elastase, and myeloperoxidase, thereby amplifying pancreatic tissue injury and systemic inflammation [8,30]. Therefore, the combination of an increased neutrophil percentage and decreased serum albumin concentration integrates two complementary pathophysiological processes, which may partly explain why NPAR demonstrated the highest predictive performance among the inflammatory indices evaluated in the present study.

Previous studies have suggested that NPAR is a useful inflammatory index reflecting systemic inflammation and may provide prognostic information in a variety of inflammatory conditions, including sepsis, spontaneous bacterial peritonitis, cardiogenic shock, chronic kidney disease, MASLD, and several malignancies [10,11,12,13,14,15]. However, evidence regarding its role in AP remains limited. Recently, Huynh et al. [17] evaluated the prognostic value of admission NPAR in 628 critically ill patients with AP admitted to the ICU. They demonstrated that higher admission NPAR values were independently associated with increased short- and long-term mortality and that NPAR outperformed most inflammatory indices in predicting mortality while showing predictive performance comparable to the BISAP score. In contrast to that study, which focused on critically ill patients and mortality outcomes, our study evaluated an unselected cohort of hospitalized patients with AP and investigated disease severity according to the revised Atlanta classification. Our findings extend the existing evidence by demonstrating that admission NPAR was independently associated with AP severity and showed the highest discriminative performance among the inflammatory indices evaluated in the present study (NLR, PLR, and SII). Although admission NPAR levels were also significantly higher in non-survivors, the relatively small number of deaths in our cohort precluded a robust evaluation of its prognostic value for mortality. Taken together with previous evidence, our findings suggest that admission NPAR may be useful for the early identification of patients at risk of developing more severe disease, while its role in mortality prediction warrants further investigation in larger prospective studies.

In addition, ROC analysis identified an admission NPAR cut-off value of 19.7 for predicting moderately severe/severe AP according to the revised Atlanta classification. Although this threshold demonstrated promising diagnostic performance in the present cohort, it should be considered exploratory and requires external validation in larger prospective studies before being adopted in routine clinical practice.

From a clinical perspective, NPAR may represent a practical and readily available inflammatory index for the early assessment of disease severity in patients with AP. Because it is derived from routinely available laboratory parameters obtained at hospital admission, it does not require additional cost, specialized testing, or extra processing time. Therefore, NPAR may assist in the early risk stratification of patients with AP and could help identify individuals who may benefit from closer clinical monitoring. However, the clinical utility of NPAR and the proposed admission cut-off value should be confirmed in prospective multicenter studies before routine clinical implementation. In addition, future studies incorporating decision curve analysis may provide further insight into the clinical utility of NPAR for guiding risk stratification and clinical decision-making in patients with AP.

This study has several limitations. First, its retrospective design and single-center setting may limit the generalizability of the findings. Second, NPAR was evaluated using only admission laboratory values, and serial measurements during hospitalization were not available. Third, although multivariable analyses were performed, the relatively limited sample size and number of outcome events may have increased the risk of model overfitting. In addition, the relatively wide distribution of inflammatory index values observed in our cohort suggests considerable biological variability, which may limit the generalizability of individual measurements despite the statistically significant associations observed. Finally, the proposed admission NPAR cut-off value was derived from the present cohort and has not been externally validated. Therefore, prospective multicenter studies with larger sample sizes are needed to validate these findings, confirm the proposed cut-off value, and further establish the clinical utility of NPAR for risk stratification in patients with AP.

## 5. Conclusions

In conclusion, admission NPAR appears to be a simple, inexpensive, and readily available inflammatory index associated with disease severity in patients with AP. In the present study, NPAR demonstrated the highest discriminative performance among the inflammatory indices evaluated (NLR, PLR, and SII) and remained independently associated with disease severity in the multivariate analysis. These findings suggest that admission NPAR may serve as a useful adjunctive tool for early risk stratification in patients with AP. However, larger prospective multicenter studies are required to validate these findings, confirm the proposed admission cut-off value, and further establish the role of NPAR in routine clinical practice.

## Figures and Tables

**Table 1:** Demographic and clinical characteristics according to AP severity.

Variable	Total *(n* = 261)	Mild AP (*n* = 159)	Moderately Severe/Severe AP (*n* = 102)	*p*-Value
Age, years	58.0 ± 18.6	56.5 ± 18.6	60.4 ± 18.4	0.095
Gender				0.853
Female	144 (55.2%)	87 (54.7%)	57 (55.8%)	
Male	117 (44.8%)	72 (45.3%)	45 (44.2%)	
Etiology of AP				0.558
Biliary	197 (75.5%)	116 (73.0%)	81 (79.4%)	
Hyperlipidemia	22 (8.4%)	12 (7.5%)	10 (9.8%)	
Alcohol	17 (6.5%)	13 (8.2%)	4 (3.9%)	
Drug-induced	6 (2.3%)	4 (2.5%)	2 (2.0%)	
Idiopathic/other	19 (7.3%)	10 (6.3%)	9 (8.8%)	
Comorbidities				
Hypertension	67 (25.6%)	37 (23.3%)	30 (29.4%)	0.268
Diabetes mellitus	48 (18.4%)	29 (18.2%)	19 (18.6%)	0.937
Chronic kidney disease	19 (7.3%)	8 (5.1%)	11 (10.7%)	0.081
Heart failure	8 (3.1%)	5 (3.1%)	3 (2.9%)	0.926
Malignancy	5 (1.9%)	3 (1.9%)	2 (2.0%)	0.966
Clinical outcomes				
Length of hospital stay, days	6 (1–69)	5 (1–22)	6.5 (2–69)	<0.001
ICU admission	33 (12.6%)	0 (0%)	33 (32.3%)	<0.001
Mortality	8 (3.1%)	0 (0%)	8 (7.8%)	<0.001

Data are presented as mean ± SD, median (min–max), or *n* (%). ICU: Intensive care unit.

**Table 2:** Baseline laboratory findings and inflammatory indices according to AP severity.

Variable	Total (*n* = 261)	Mild AP (*n* = 159)	Moderately Severe/Severe AP (*n* = 102)	*p*-Value
Laboratory parameters				
ALT (U/L)	93 (5–1386)	92 (5–751)	93 (7–1386)	0.119
AST (U/L)	94 (8–1700)	84 (8–1010)	129 (11–1700)	0.039
LDH (U/L)	301 (128–1798)	270 (128–954)	342 (142–1798)	<0.001
Glucose (mg/dL)	124 (56–514)	114 (65–433)	144 (56–514)	<0.001
Urea (mg/dL)	37 (10.5–127)	32.3 (10.5–98)	44.3 (14–127)	<0.001
Creatinine (mg/dL)	0.8 (0.3–3.7)	0.8 (0.3–1.4)	0.8 (0.4–3.7)	0.573
Amylase (U/L)	931 (41–6701)	769 (41–5907)	1148 (47–6701)	0.061
Lipase (U/L)	1620 (55–18,896)	1351 (55–18,896)	1978 (63–14,500)	0.047
WBC (×10^9^/L)	10.5 (3.5–29.9)	9.2 (3.5–28.6)	12.5 (5.0–29.9)	<0.001
Neutrophils (×10^9^/L)	7.8 (2.5–27.2)	6.7 (2.5–26.3)	10.7 (2.8–27.2)	<0.001
Neutrophil percentage (%)	75.9 (34.8–94.6)	71.8 (42.7–93.7)	82.2 (34.8–94.6)	<0.001
Lymphocytes (×10^9^/L)	1.5 (0.3–4.8)	1.7 (0.3–4.8)	1.2 (0.3–4.0)	<0.001
Hematocrit (%)	40.0 ± 5.2	39.6 ± 4.6	40.5 ± 6.2	0.191
Platelet count (×10^9^/L)	237 (111–471)	233 (111–471)	243 (193–466)	0.738
Albumin (g/dL)	3.8 ± 0.5	4.0 ± 0.4	3.6 ± 0.62	<0.001
CRP (mg/dL)	10.3 (1–300)	8.4 (1–209)	12.6 (1–300)	0.322
Inflammatory indices				
NLR	5.7 (1–38.9)	3.5 (1–38.9)	10.2 (1.9–36.2)	<0.001
NPAR	19.8 (9.5–46.8)	17.6 (9.5–39.2)	22.5 (13.4–46.8)	<0.001
PLR	160.7 (50.5–880)	141.2 (50.5–838)	203 (67–880)	<0.001
SII	1275 (245–15,136)	956 (245–10,140)	2249 (499–15,136)	<0.001

Data are presented as mean ± SD or median (min–max). ALT: Alanine aminotransferase, AST: Aspartate aminotransferase, LDH: Lactate dehydrogenase, WBC: White blood cell count, CRP: C-reactive protein, NLR: Neutrophil-to-lymphocyte ratio, NPAR: Neutrophil percentage-to-albumin ratio, PLR: platelet-to-lymphocyte ratio, SII: systemic immune inflammation index.

**Table 3:** Comparison of the predictive performance of inflammatory indices for moderately severe/severe AP according to the revised Atlanta classification and BISAP score.

	Atlanta (Moderately Severe/Severe)		BISAP ≥ 3	
İnflammatory Indices	AUC (95% CI)	*p*-Value	AUC (95% CI)	*p*-Value
NPAR	0.808 (0.747–0.854)	<0.001	0.841 (0.746–0.897)	<0.001
NLR	0.754 (0.696–0.813)	<0.001	0.774 (0.693–0.855)	<0.001
SII	0.758 (0.700–0.816)	<0.001	0.787 (0.707–0.867)	<0.001
PLR	0.653 (0.583–0.722)	<0.001	0.668 (0.557–0.779)	0.002

**Table 4:** Predictive performance of admission NPAR for AP severity.

Reference	Cut-Off	AUC (95% CI)	Sensitivity (%)	Specificity (%)	PPV (%)	NPV (%)	*p*-Value
Atlanta classification (moderately severe/severe AP)	19.7	0.808 (0.755–0.860)	81.6	71.3	63.1	85.1	<0.001

AUC: area under the curve; CI: confidence interval; PPV: positive predictive value; NPV: negative predictive value.

**Table 5:** Univariate and Multivariate Logistic Regression Analyses for predicting moderately severe/severe AP.

Parameter	Univariate Analysis			Multivariate Analysis		
	OR	95% CI	*p*	OR	95% CI	*p*
Age	1.012	0.998–1.025	0.095	0.968	0.949–0.988	0.002
WBC	1.259	1.169–1.356	<0.001	1.133	1.041–1.233	0.004
Urea	1.036	1.020–1.052	<0.001	1.017	0.998–1.037	0.076
NPAR	1.315	1.220–1.417	<0.001	1.279	1.174–1.393	<0.001

OR: odds ratio; CI: confidence interval; WBC: white blood cell count; NPAR: neutrophil percentage-to-albumin ratio.

## Data Availability

The data supporting the conclusions of this article will be made available by the corresponding author on request.

## Supplementary Materials

The following supporting information can be downloaded at: https://www.mdpi.com/article/10.3390/biomedicines14071543/s1. Table S1: Subgroup analysis of admission NPAR according to AP severity.
