# Supplementary material for: Evaluation of the Admission Neutrophil Percentage-to-Albumin Ratio for Predicting the Severity of Acute Pancreatitis
Source: Biomedicines. 2026 Jul 9;14(7):1543. doi: 10.3390/biomedicines14071543 (PMC13404601; doi:10.3390/biomedicines14071543)
Supplement: Supplementary file 1 [file biomedicines-14-01543-s001.zip › biomedicines-4398124-supplementary.pdf]

Table S1. Subgroup analysis of admission NPAR according to acute pancreatitis severity

**Subgroup**	**Mild AP**	**Moderately severe/severe AP**	***p* value**
Male	17.20 (9.53–30.34)	21.36 (14.95–39.61)	<0.001
Female	17.76 (10.64–37.47)	24.13 (13.39–46.83)	<0.001
Age <60 years	16.51 (9.53–25.65)	20.53 (13.39–31.21)	<0.001
Age ≥60 years	19.06 (12.11–37.47)	25.91 (16.99–46.83)	<0.001
No comorbidity	17.11 (9.53–30.34)	21.73 (15.17–46.83)	<0.001
≥1 comorbidity	18.57 (11.98–37.47)	25.38 (13.39–39.61)	<0.001

Data are presented as median (minimum–maximum).
